# Observational study of endoluminal mural thrombotic apposition in popliteal artery aneurysm stenting and its relationship with stent-graft geometrical features

**DOI:** 10.3389/fcvm.2023.1176455

**Published:** 2023-08-07

**Authors:** Giovanni Spinella, Michele Conti, Marco Magliocco, Fabio Riccardo Pisa, Alice Finotello, Martina Pulze, Giovanni Pratesi, Giuseppe Cittadini, Giancarlo Salsano, Bianca Pane

**Affiliations:** ^1^ Department of Surgical and Integrated Diagnostic Sciences, University of Genoa, Genoa, Italy; ^2^ UOC Clinica di Chirurgia Vascolare ed Endovascolare, IRCCS Ospedale Policlinico San Martino, Genoa, Italy; ^3^ Department of Civil Engineering and Architecture, University of Pavia, Pavia, Italy; ^4^ IRCCS MultiMedica, Milan, Italy; ^5^ Department of Radiology, IRCCS Ospedale Policlinico San Martino, Genoa, Italy

**Keywords:** intrastent thrombosis, peripheral stenting, endovascular treatment, popliteal aneurysm, popliteal aneurysm treatment

## Abstract

**Introduction:**

The development of intrastent thrombosis is one of the mechanisms related to medium- to long-term failure of endovascular treatment of popliteal artery aneurysm. The present study aims to investigate possible links between the development of endoluminal mural thrombotic apposition in the stented zone (EMTS) with both geometrical features of stent-graft(s) and time of follow-up.

**Methods:**

Patients with popliteal artery aneurysm who underwent endovascular treatment were recruited during the follow-up period. Segmentation of computed tomography angiography scan was performed to detect femoropopliteal artery lumen, leg bones, EMTS, and stent-graft(s). The following parameters were assessed: number, diameter, and length of stent-graft(s); and shape, volume, and length of thrombotic apposition within the stent(s). The spiral shape of the thrombotic apposition was evaluated as well.

**Results:**

Eighteen male patients were recruited in the study. EMTS was observed in 13 of them (72%) during the follow-up analysis. An average of 1.8 ± 0.79 stents-grafts were implanted per patient with a median diameter and length of 6.2 (1.9) mm and 125 (50) mm, respectively. The percentage of the stent length where EMTS was present was 42.1 on average (interquartile range: 42.4%) with a mean volume of 206.8 mm^3^. A positive correlation was found between the length and volume of EMTS (R-squared = 0.71, *p* < 0.01). Moreover, EMTS had a helical shape in 8/13 patients, with 4/5 with counterclockwise rotation with stent-grafts in the left leg and 3/3 with clockwise direction treated in the right leg. A higher frequency of EMTS was observed in patients with longer follow-up and higher risk factors, as well.

**Conclusions:**

EMTS is observed in most of the patients under analysis, especially in those with medium- to long-term follow-up. The pattern of such EMTS follows a helical shape having a direction that depends on which leg, right or left, is treated. Our results suggest a close surveillance of popliteal aneurysm stenting by follow-up examinations to control the onset and progression of EMTS.

## Introduction

Nowadays, endovascular treatment (ET) of popliteal artery aneurysms (PAAs) is recommended in patients with limited life expectancy and adequate landing zones with adequate outflow vessels (1).

Literature data show that ET offers significant advantages in terms of shorter average hospital stay and better post-operative recovery when compared with open surgical repair; although the follow-up results show that the primary patency of open surgical repair is better than ET, the medium- to long-term efficacy of ET is still a matter of debate (2).

Infolding, dislocation, stent fracture, endoleaks, and compression are considered the main causes of endovascular treatment failure. Moreover, recently, some authors have indicated stent-graft thrombosis—related to endoluminal mural thrombotic apposition in the stented zone (EMTS)—as one of the causes of ET failure (3); but, unfortunately, such indications are referred to as single case studies, calling for an analysis of a large number of cases to buttress the findings.

Furthermore, a recent study by Ichihashi et al. (4) sought to elucidate the clinical impact and prognosis of stent-graft thrombosis, showing that this unfavorable outcome was present in 13% of the population (i.e., 1,215 patients). Unfortunately, the authors did not report any indication of the onset and progression of EMTS. Indeed, to the best of our knowledge, there are no prior hypotheses regarding the formation of EMTS in patients treated with endovascular procedures for PAA. Although it is known that the biggest advantage of the covered stent-graft is that in-stent intimal hyperplasia theoretically does not occur because of the presence of the Enhancing expanded poly(tetrafluoroethylene) (ePTFE) membrane (4), we have observed EMTS. Further investigations leveraged by the computer-based investigation of local hemodynamics (5) are required to explain the onset and progression mechanisms of EMTS.

Moreover, an analysis of the follow-up data after treatment of popliteal aneurysm shows lower primary patency for endovascular treatment compared with open repair (6, 7). However, the limb salvage rate is not different between the two groups (8); this could be explained by the slow occurrence of stent-graft thrombosis during follow-up, thus allowing the possibility of compensation circles supporting the limb salvage without the need for reinterventions. Therefore, the detection, quantification, and analysis of EMTS could further explain this aspect.

To this aim, this study intends to assess, in a cohort of patients treated endovascularly for PAA, the presence of EMTS by conducting an analysis of follow-up computed tomography angiography (CTA) scan, highlighting, at the same time, a possible correlation between EMTS and stent-graft(s) geometrical features.

## Materials and methods

Patients with PAA who underwent ET were recruited retrospectively for an imaging study (9). Signed informed consent was obtained from all patients, and all procedures were performed in accordance with the Declaration of Helsinki and approved by the local ethics committee (GR-2018-12368376).

For patients deemed eligible for endovascular PAA treatment, the length of the proximal and distal landing zones should be at least 15 mm, with the dilatation artery being relatively straight and free from stenosis or off target and with at least one run-off vessel. Moreover, the choice of the treatment should be based on the comorbidities and preferences of patients.

The inclusion criteria were previous endovascular treatment, availability of follow-up CTA scan, and age older than 45  years. Exclusion criteria were contraindications to CTA, inability to give signed informed consent, and previous peripheral surgical or endovascular procedures.

All patients had undergone endovascular treatment for popliteal aneurysm. The treatment had been planned following a study by Spinella et al. (9). Briefly, an appropriate proximal and distal landing zone was identified by evaluating the preoperative CTA. Endovascular treatment was performed under spinal anesthesia by gaining surgical access to the proximal superficial femoral artery. One or more Viabahn-covered stents (W.L. Gore & Associates, Flagstaff, AZ, USA) were positioned according to endovascular planning. All evaluated patients were administered double antiplatelet therapy (clopidogrel 75 mg/d and acetylsalicylic acid 100 mg/d).

As previously reported, during follow-up, one contrast-enhanced CTA of the lower limbs was acquired in each recruited patient in addition to the routine Duplex ultrasound (DU). Post-operative CTA images of each patient were collected and anonymized. The mean follow-up period was 14 months, as reported in Table 1.

**Table:** Data overview of patients.

	Total	EMTS	NO-EMTS
Patients	18	13	5
Median age	74	74	73
Mean follow-up (months)	14.3	16.7	8
Distal landing zone			
P1	1 (5.5%)	0	1 (20%)
P2	4 (22.2%)	2 (15.4%)	2 (40%)
P3	13 (72.2%)	11 (84.6%)	2 (40%)
*N* stent			
1	8	5	3
2	6	5	1
3	4	3	1
Run-off vessels			
1	2 (11.1%)	2 (15.4%)	0
2	7 (38.8%)	5 (38.4%)	2 (40%)
3	9 (50%)	6 (46.2%)	3 (60%)
Proximal landing zone diameter (mean)	6.7	6.6	6.7
Distal landing zone diameter (mean)	5.4	5.2	6.0
Δ diameter (%)	18.5	21.1	11.4
Cardiovascular risk factors			
Smoke	1	1 (7.7%)	0 (0%)
Hypertension	13	12 (92%)	1 (20%)
Dyslipidemia	6	5 (38.4%)	1 (20%)
Ischemic heart disease	5	5 (38.4%)	0

For each patient included in the study, the following parameters were assessed: number of implanted stent-graft(s); diameter and length of the stent-graft(s); number of run-off patent vessels; the mean diameter of the proximal and distal edge of the stent and the popliteal artery below the stent segment; % variations between proximal and distal diameter (Δ diameter, %); and shape, volume, and length of EMTS, if present.

Measurements of peak systolic velocity (PSV) in the popliteal artery and the flow rate in the distal landing zone were extracted from the post-operative DU.

All measurements were performed for all recruited patients, who were divided in two subgroups on the basis of the presence or absence of EMTS (i.e., EMTS vs. NO-EMTS).

Segmentation of the CTA scan was performed by using open-source software ITK-Snap (10) to detect the following: lumen of Femoro-Popliteal Artery (FPA) from the femoral artery bifurcation to the popliteal artery bifurcation, leg bones, EMTS, and the stent-graft(s). Finally, where present, EMTS was virtually removed to define a surrogate model of the intrastent lumen without thrombus; such a model is necessary to estimate the actual stent-graft diameter to be compared with its nominal value.

According to Spinella et al. (9), the result of the segmentation has been exported as stereo-lithographic files (.stl) to be elaborated by the open-source application ParaView (11) to clip the stented region and compute the lumen centerline using the Vascular Modeling ToolKit (VMTK) (12). Such an analysis was repeated for each stent-graft in case of multiple implants. Finally, for each stent-graft, the centerline was elaborated to calculate the percentage of the stent in which EMTS was present. In particular, the analysis of the cross-sectional lumen diameter along the centerline was used to detect the presence of thrombosis: for each point of the centerline, the difference in the diameter between the actual lumen diameter with the corresponding value obtained with the surrogate model without thrombosis was computed. A difference higher than 0.1 mm was considered an indicator of the presence of EMTS.

As already stated, the mean diameter of the intrastent lumen without the thrombus was also computed in order to compare the actual stent diameter with its nominal value; the volume of the thrombus was computed as well and it was calculated on the basis of the segmentation. In addition, a difference of stent graft diameter between proximal and distal landing zone has been carried out.

**Figure 1:**
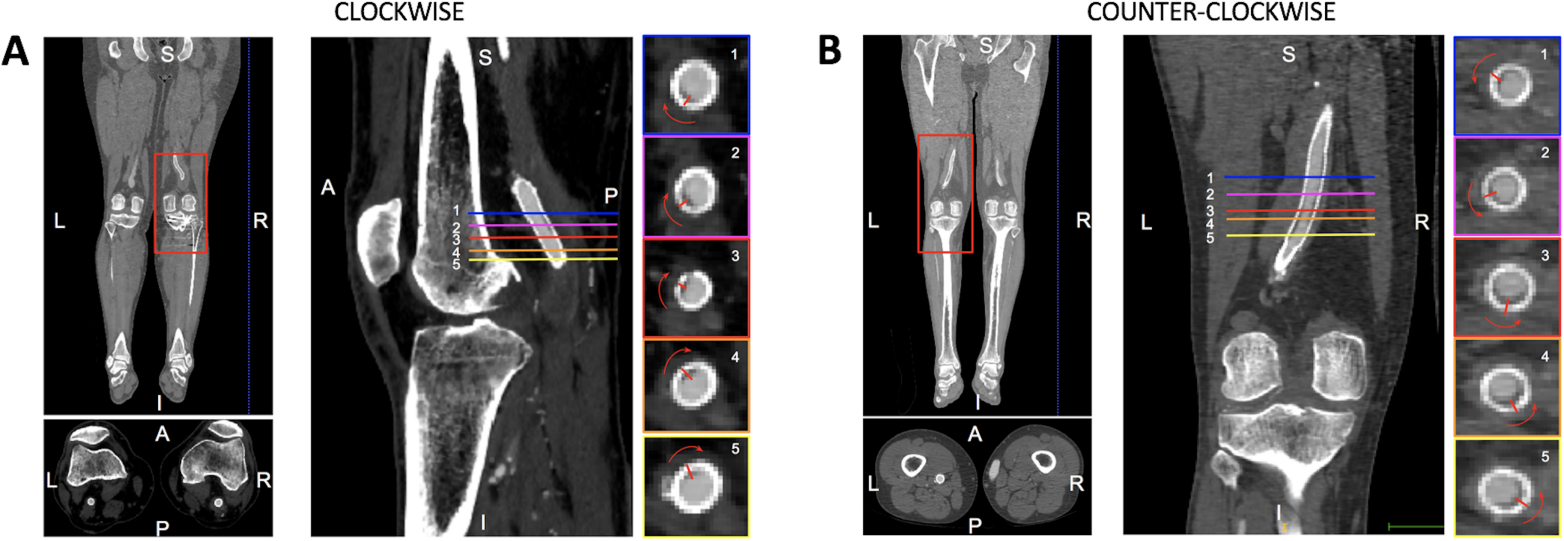
Illustrative representation of the CTA analysis to assess the helical shape of the thrombosis and its rotation along the femoropopliteal axis. This is an analysis of the right leg showing a clockwise rotation of the thrombosis as the axial slices shift from top (1) to bottom (5).

The spiral shape of the stent-graft thrombus was assessed through a visual inspection of CTA images and 3D surfaces by an expert radiologist. The direction of rotation of EMTS was assessed in each CTA image by analyzing the CTA slices from top to bottom and categorized as clockwise or counterclockwise, as shown in Figure 1.

### Statistical analysis

Continuous variables are expressed as median and interquartile range (IQR) or mean and standard deviation, where appropriate. Linear regression was performed using the least squares method to study the correlation between the parameters. An R-squared value of >0.1 and a *p*-value of <0.05, which was obtained from the *F*-test, were considered statistically significant in the assessment of the resulting models. All analyses were performed using MATLAB software v. R2021a (Mathworks Inc., Natick, MA, USA).

## Results

Between January 2017 and January 2021, 23 patients underwent endovascular treatment for popliteal aneurysm at our unit. Among them, 18 patients were included in the study according to the proposed inclusion and exclusion criteria; of these 18, 13 (72.2%) developed EMTS.

Patient characteristics and risk factors are reported in Table 1 both for the total group of patients and the two subgroups (EMTS/NO-EMTS). We recall that at the time of evaluation, all patients were administered dual antiplatelet therapy (clopidogrel 75 mg/d and acetylsalicylic acid 100 mg/d) and no patient was administered anticoagulant therapy.

All patients were male, and the median age was 73.5 years (IQR: 10).

The median of the overall follow-up period was 12 months (IQR: 18). During the follow-up period, although EMTS was observed in most patients, no stent occlusion was observed. The median value of follow-up in the EMTS group was higher than that in the NO-EMTS group (13 vs. 5 months).

The P3 distal landing zone was observed in most patients under investigation (*n* = 13, 72.2%); such a distribution was observed for the EMTS group as well.

Eight patients were treated with one stent, six patients with two stents, and four patients received three stents, totaling 32 stents. Most of the patients were treated with two or three stents (56%) and the same trend was observed for the EMTS group, while 60% of the patients for whom EMTS was absent were treated with one stent.

As shown in Table 1, in half of the cases, the number of run-off is 3 and there is no evidence on the distribution of the number of run-off vessels in the subgroups.

The median of nominal diameter and length of the stented segments were 8 mm (IQR: 1) and 125 mm (IQR: 50), respectively. The measured actual stent diameter was smaller than the nominal one in all patients, with a median difference of 2.2 mm (IQR: 0.7 mm). As shown in Table 1, there is no difference between the proximal landing zone diameter in the subgroups (*p* = 0.1), while the distal diameter in the NO-EMTS group is higher, even if no statistical significance is reached (*p* = 0.07). It is worth noting that the percentage variations between the proximal and the distal diameter are lower in the NO-EMTS group than in the EMTS group, suggesting that tapering was higher in the EMTS group than in the other group.

We observed hypertension, dyslipidemia, and ischemic heart disease in 13, six, and five patients, respectively. The percentage of EMTS patients with at least one of these factors is generally higher than in the NO-EMTS group. According to the results obtained, there is a statistically significant difference at a 1% probability level for patients with hypertension belonging to the EMTS/NO-EMTS group.

Details of the stent-graft(s) and EMTS characteristics for each patient and stent(s) are provided in Supplementary Table S1.

The median length of EMTS per stent was 47.8 mm (IQR: 83.4 mm). The thrombotic apposition constituted 42.1% (IQR: 42.4%) of the total length of the stent along the centerline.

The volume of EMTS was 206.8 mm^3^ (IQR: 823.6 mm^3^).

**Figure 2:**
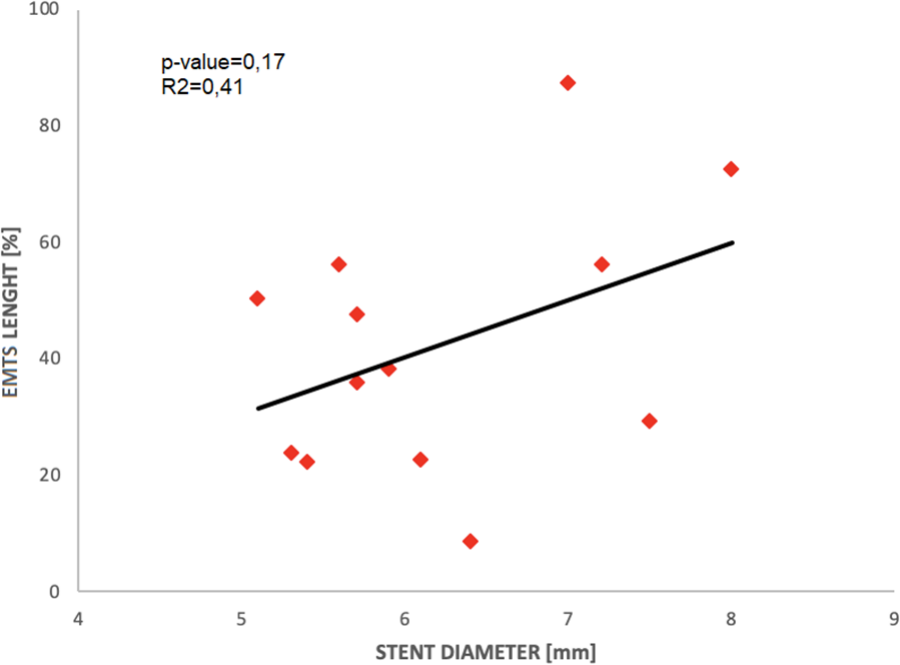
Median stent-graft diameter vs. percentage of EMTS length.

As shown in Figure 2, there is no statistically significant correlation between stent diameter and the percentage of EMTS volume or EMTS length, even if a positive trend is observable; that is, EMTS length (as a percentage of the stent length) increases with the stent diameter: this suggests that proximal stents are more prone to thrombus onset and progression because the diameter is larger and unfavorable fluid dynamics conditions are present, as shown in (5). Moreover, there is a significant positive correlation between EMTS length and volume (R-squared = 0.71, *p* < 0.01).

An analysis of the shape of the thrombotic apposition revealed the presence of portions of thrombosis with a helical shape in 8/13 (61.5%) patients, a counterclockwise rotation in 4/5 (80%) of patients with stent-grafts in the left leg, and a clockwise rotation in 3/3 (100%) patients treated in the right leg. The results for single patient cases are reported in Supplementary Table S2.

**Figure 3:**
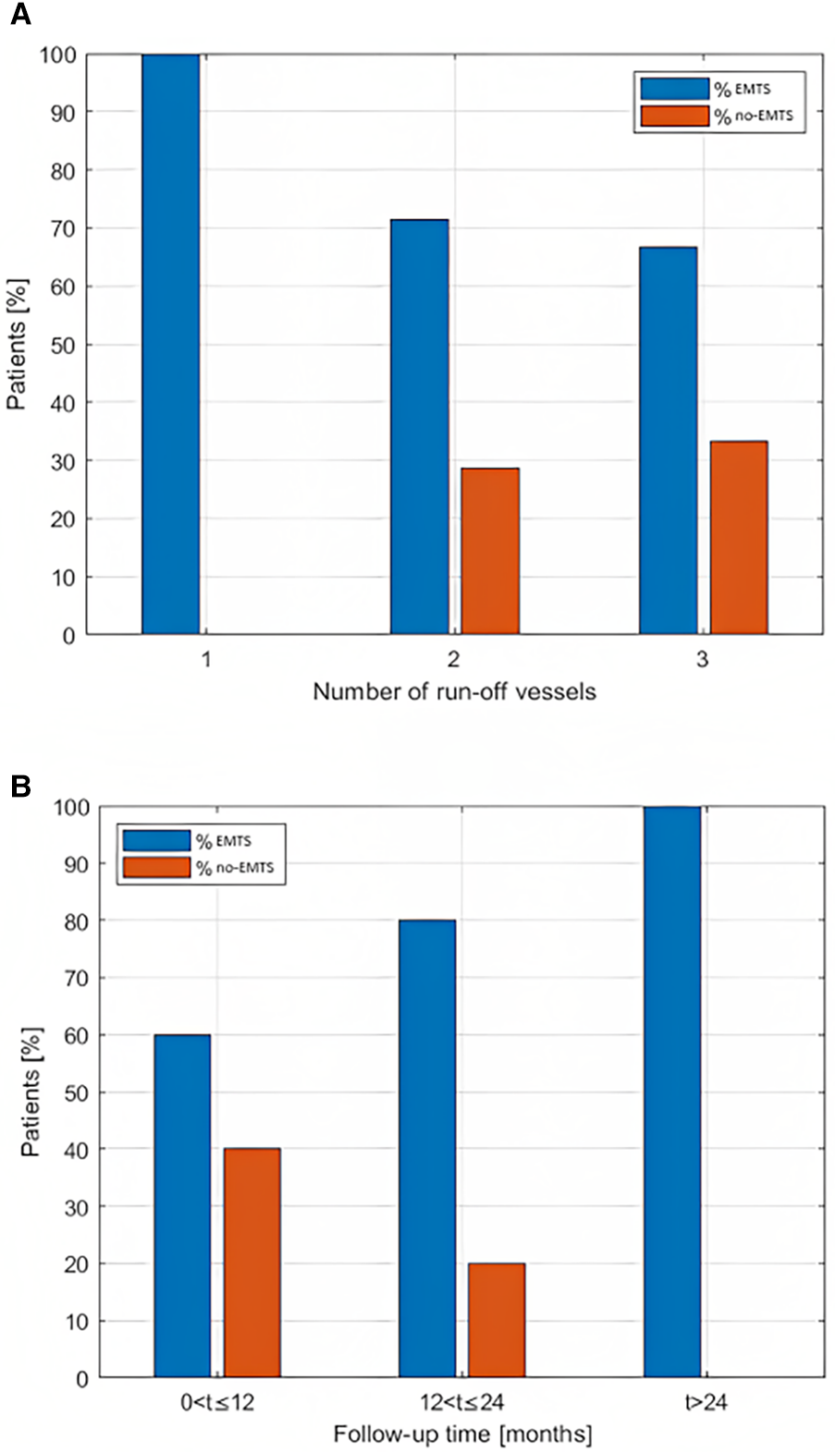
(**A**) Percentage of patients (EMTS/NO-EMTS) in relation to the number of pervious run-off vessels. (**B**) Percentage of patients (EMTS/NO-EMTS) grouped according to follow-up duration.

The percentages of patients for the EMTS and NO-EMTS groups categorized by the run-off vessel number (1, 2, or 3) are shown in Figure 3A.

With regard to the presence of EMTS in relation to the follow-up duration, Figure 3B shows that the percentage of EMTS patients increases as a function of follow-up time. In particular, we observed that 60% of patients within 12 months of follow-up (*n* = 6) showed EMTS; such a percentage increased to 80 when the follow-up ranged from 1 to 2 years (*n* = 4), while all patients whom we observed after 24 months of follow-up showed EMTS (*n* = 3).

In the post-operative DU, PSV, and flow rate were 560 mm/s (196) and 0.3 L/min (0.31) for the total group of patients. In the two subgroups, the median flow rate was 0.35 L/min (0.27) for EMTS patients and 0.26 L/min (1.26) for NO-EMTS patients, respectively (*p* = 0.1).

## Discussion

A retrospective follow-up analysis of patients who underwent endovascular treatment for PAA was performed with the aim of providing information about EMTS.

Unfortunately, data from the literature are scarce and do not provide a comparison for this type of analysis. This issue should be addressed when the modalities of routine follow-up examinations are prepared, which usually refer only to Doppler Ultrasound as recently recommended by the guidelines (1). Some authors have already highlighted how DU fails to identify endovascular treatment failures (13). For this reason, a follow-up CT scan for PAA after ET could be considered. Previously, some authors have already reported the use of the follow-up CTA scan to evaluate the shrinkage of the aneurysmal sac after ET (14).

Our results have highlighted how performing a follow-up CTA scan every 12 months can allow us to identify the presence of EMTS, which does not always require treatment but should be monitored during frequent follow-ups. Moreover, this follow-up protocol might allow us to identify the cause of endovascular treatment failure. In fact, despite its short-term advantages, ET has failed to become the treatment of choice for PAA (1). EMTS could be a possible cause of stent occlusion during follow-up, and our results add evidence in the form of data on the possible causes of long-term endovascular treatment failure.

With regard to EMTS, our results have shown how a helical shape is observed in most of the analyzed patient cases. We have also identified the direction of rotation, which is in the counterclockwise sense in 80% of patients stented in the left leg and in the clockwise sense in all patients stented in the right leg.

Recently, some authors have reported a case of a patient in whom a helical shape of EMTS was observed during follow-up angiographic examination after popliteal artery aneurysm ET (3).

Our results show that EMTS is related to follow-up duration, even if thrombosis occurs in the first few months after treatment, although this is not clinically relevant.

This information is important because it shows, for the first time, how EMTS increases in volume over time and how such data can be relied upon to identify the cause of the lower patency of endovascular treatment over time compared with open surgical treatment. In fact, to date, different causes have been identified to justify the failure of endovascular treatment, such as infolding, endoleak, or stent-graft dislocation. Thanks to a proper selection of patients to undergo endovascular treatment, many of these causes can be minimized today; but in spite of this, cases of ET failure can be found (15).

Moreover, although it was not possible to observe a strong positive correlation between mean stent diameter size and the presence of thrombosis, we observed EMTS in stents with a larger diameter (i.e., equal to or greater than 8 mm), while in stents with a smaller diameter, we found no presence of EMTS.

These data can be correlated with each other taking into account the flow rate inside the stent. In fact, for a given flow rate in the stents with a larger diameter, blood velocity could be lower and this could favor a turbulent flow that could lead to EMTS formation, while in the stents with a smaller diameter, blood velocity remains higher and the flow more laminar, and therefore, the formation of EMTS is less likely. This hypothesis has been recently verified through fluid dynamics simulations in a single case study (5). Another consideration is that what develops inside the stent is not correlated to the presence of one or three run-off vessels. In addition, the position of the distal landing zone in P3 promotes thrombus development because the stented area will be larger.

Given such considerations, our results may have clinical relevance and the planning of endovascular treatment may be modified. Currently, taking into consideration the diameter of the proximal and distal landing zones and the length to be covered, one or more covered stents are positioned in order to exclude PAA. Nowadays, we do not have conical covered stents, and therefore, if there are different diameters of the proximal and distal landing zones, two or three stents with different diameters are positioned. As shown in Table 1, EMTS patients also have a greater variation in diameter, between proximal and distal stents, than patients who do not develop EMTS. Taking these findings into account, preference should be given to the use of covered stents with smaller diameters (when possible) with longer lengths and to ensure proximal sealing with shorter stents but with larger diameters in order to reduce the risk of EMTS.

Finally, adherence to follow-up antiplatelet therapy that has a role in conditioning primary patency could also play a role in helping us explain EMTS. In our clinical experience, we have discovered that all analyzed patients have undergone double antiplatelet therapy since undergoing ET.

The main limitation of the study is the small number of patients analyzed. However, the prevalence of PAA is low, and of these PAA patients, only a small percentage of them undergo endovascular treatment and develop EMTS.

Another limitation is related to the non-homogeneity of the follow-up period. Future studies with prospectively enrolled patients will overcome this issue. Finally, the analysis of a larger population, favoring statistical analysis, can strengthen the proposed observations.

## Conclusions

In conclusion, our results have shown that within the covered stents positioned for the ET of PAA, EMTS is observed during follow-up, and it is observed in different follow-up lengths, particularly in patients with a longer follow-up period. Furthermore, it has been observed that EMTS has a helical shape with either a clockwise or a counterclockwise direction, which depends on which leg, right or left, is treated. The exact underlying cause of this phenomenon has not yet been fully understood; accordingly, future perspectives of the present study foresee Computational Fluid Dynamics (CFD) simulations (5) in order to highlight the role of local arterial hemodynamics in the pattern of EMTS. Our data demonstrate how different factors are involved in the development of EMTS, which can affect the primary patency of a stent-graft. In the light of this, prospective studies with more evaluated patients may provide guidance on how to change the schedule of endovascular treatment to improve treatment outcomes, especially in long-term follow-up.

## Data Availability

The raw data supporting the conclusions of this article will be made available by the authors without undue reservation upon request.
