# Supplementary material for: Observational study of endoluminal mural thrombotic apposition in popliteal artery aneurysm stenting and its relationship with stent-graft geometrical features
Source: Front Cardiovasc Med. 2023 Aug 7;10:1176455. doi: 10.3389/fcvm.2023.1176455 (PMC10441546; doi:10.3389/fcvm.2023.1176455)
Supplement: Supplementary file 1 [file Table1.doc]

***Supplementary Material***

**Observational study of endoluminal mural thrombotic apposition in popliteal artery aneurysm stenting and its relationship with stent-graft geometrical features**

**Giovanni Spinella**^†^**, Michele Conti**^†^**, Marco Magliocco, Fabio Riccardo Pisa, Alice Finotello, Martina Pulze, Giovanni Pratesi, Giuseppe Cittadini, Giancarlo Salsano, Bianca Pane^*^**

*** Correspondence:** Bianca Pane: [bianca.pane@unige.it](mailto:bianca.pane@unige.it)

**Supplementary Table I.** Results of geometrical analysis.

| **ID patient** | **Nominal stent diameter [mm]** | **Actual mean stent graft diameter [mm]** | **EMTS length [mm]** | **EMTS length [%]** | **EMTSvolume [mm^3^]** | **EMTS volume [%]** | **Proximal landing zone diameter [mm]** | **Distal landing zone diameter [mm]** | **Δ_diameter (proximal-distal) [%]** | **Poplitea diameter [mm]** |
| --- | --- | --- | --- | --- | --- | --- | --- | --- | --- | --- |
| 01_S1 | 11 | 8.4 | 5.7 | 27.6 | 14.5 | 1.1 | 8,5 | 5,6 | 34 | 7,4 |
| 01_S2 | 8 | 5.5 | 119.4 | 49.1 | 838.1 | 13.2 |
| 02 | 8 | 5.6 | 113.3 | 56.1 | 574.9 | 11.4 | 5,6 | 5,5 | 1,8 | 4,9 |
| 03_S1 | 11 | 8.8 | 40.8 | 75.9 | 1094.1 | 28.8 | 8,3 | 6,2 | 25,3 | 9 |
| 03_S2 | 10 | 7.4 | 22.8 | 34.3 | 96.0 | 3.2 |
| 03_S3 | 9 | 6.3 | 0.0 | 0.0 | 0.0 | 0 |
| 04_S1 | 10 | 7.9 | 126.0 | 100.0 | 1826.0 | 27.3 | 7,2 | 3,8 | 47,2 | 5,5 |
| 04_S2 | 8 | 5.7 | 6.4 | 6.7 | 26.5 | 1 |
| 04_S3 | 6 | 3.6 | 0.0 | 0.0 | 0.0 | 0 |
| 05 | 10 | 8.0 | 102.3 | 72.7 | 1476.5 | 18.6 | 7,2 | 7,1 | 1,4 | 11,4 |
| 06 | 8 | 5.1 | 128.6 | 50.4 | 1397.5 | 23 | 4,4 | 2,6 | 40,9 | 4,7 |
| 07_S1 | 9 | 6.3 | 57.2 | 51.9 | 386.9 | 9.7 | 5 | 4,2 | 16 | 6,4 |
| 07_S2 | 7 | 4.5 | 0.0 | 0.0 | 0.0 | 0 |
| 08 | 9 | 7.0 | 86.8 | 87.3 | 1111.8 | 25.3 | 6,6 | 5,5 | 16,7 | 7,7 |
| 09_S1 | 10 | 7.3 | 25.6 | 42.1 | 189.4 | 6.7 | 7 | 6 | 14,3 | 7,7 |
| 09_S2 | 9 | 7.1 | 89.8 | 61.7 | 697.7 | 11.3 |
| 010 | 8 | 5.3 | 57.9 | 23.8 | 300.9 | 4.9 | 5,5 | 5 | 9,1 | 8 |
| 11-S1 | 9 | 6.8 | 47.8 | 48.3 | 206.8 | 5.9 | 6,8 | 5,1 | 25 | 8,3 |
| 11-S2 | 9 | 6.8 | 83.9 | 61.6 | 472 | 8.6 |
| 11-S3 | 7 | 5.1 | 54.3 | 22.2 | 150.2 | 2.8 |
| 12-S1 | 9 | 7.6 | 14 | 19.2 | 2.7 | 0.03 | 7,5 | 5,4 | 28 | 7,5 |
| 12-S2 | 7 | 5.4 | 0 | 0 | 0 | 0 |
| 13 | 8 | 6,1 | 31.7 | 22.5 | 139 | 3.2 | 6,6 | 5,5 | 16,7 | 8,4 |
| 14 | 11 | 9.6 | - | - | - | - | 10,1 | 9,3 | 7,9 | 9,5 |
| 15 | 7 | 5.1 | - | - | - | - | 4,5 | 4,1 | 8,9 | 5,5 |
| 16 | 8 | 5.7 | - | - | - | - | 6,4 | 5,8 | 9,4 | 5,5 |
| 17-S1 | 8 | 5.8 | - | - | - | - | 5,8 | 4,1 | 29,3 | 6,5 |
| 17-S2 | 7 | 4.5 | - | - | - | - |
| 18-S1 | 8 | 6.6 | - | - | - | - | 6,7 | 6,6 | 1,5 | 7,4 |
| 18-S2 | 8 | 6.2 | - | - | - | - |
| 18-S3 | 9 | 6.2 | - | - | - | - |
| **Median** | 8 | 6,2 | 47,8 | 42,1 | 206,8 | 5,9 |  |  |  |  |
| **IQR** | 1 | 1,9 | 83,4 | 42,4 | 823,6 | 12,2 |  |  |  |  |

Legend: S1: proximal stent; S2: distal stent in case of two stents; S3: distal stent in case of three stents.
