# Supplementary material for: Observational study of endoluminal mural thrombotic apposition in popliteal artery aneurysm stenting and its relationship with stent-graft geometrical features
Source: Front Cardiovasc Med. 2023 Aug 7;10:1176455. doi: 10.3389/fcvm.2023.1176455 (PMC10441546; doi:10.3389/fcvm.2023.1176455)
Supplement: Supplementary file 2 [file Table2.doc]

***Supplementary Material***

**Observational study of endoluminal mural thrombotic apposition in popliteal artery aneurysm stenting and its relationship with stent-graft geometrical features**

**Giovanni Spinella**^†^**, Michele Conti**^†^**, Marco Magliocco, Fabio Riccardo Pisa, Alice Finotello, Martina Pulze, Giovanni Pratesi, Giuseppe Cittadini, Giancarlo Salsano, Bianca Pane^*^**

*** Correspondence:** Bianca Pane: [bianca.pane@unige.it](mailto:bianca.pane@unige.it)

**Supplementary Table II.** Patient data, including analyses of the helix shape of thrombosis.

| **Patient ID** | **Number of stents** | **Stent diameter-stent length [mm]**  **(proximal, intermediate, distal)** | **Number of run-off vessels** | **Follow-up duration [months]** | **Stented leg** | **EMTS** | **Helical shape** | **Sense of rotation** |
| --- | --- | --- | --- | --- | --- | --- | --- | --- |
| 01 | 2 | 11-100,8-250 | 2 | 13 | Right | Yes | No | - |
| 02 | 2 | 8-100,8-150 | 2 | 20 | Left | Yes | Yes | Counter-  clockwise |
| 03 | 3 | 11-100,10-100,9-100 | 2 | 15 | Left | Yes | Yes | Counter-  clockwise |
| 04 | 3 | 10-150,8-150,6-150 | 1 | 1 | Right | Yes | Yes | clockwise |
| 05 | 1 | 10-150 | 3 | 57 | Left | Yes | Yes | Counter-  clockwise |
| 06 | 1 | 8-250 | 3 | 12 | Right | Yes | No | - |
| 07 | 2 | 9-150,7-150 | 3 | 1 | Left | Yes | Yes | Counter-  clockwise |
| 08 | 1 | 9-100 | 2 | 5 | Left | Yes | No | - |
| 09 | 2 | 10-100,9-150 | 3 | 1 | Left | Yes | Yes | clockwise |
| 10 | 1 | 8-250 | 3 | 38 | Right | Yes | Yes | clockwise |
| 11 | 3 | 9-100, 9-150, 7-150 | 1 | 24 | Right | Yes | Yes | clockwise |
| 12 | 2 | 9-100, 7-100 | 3 | 26 | Left | Yes | No | - |
| 13 | 1 | 8-150 | 2 | 4 | Left | Yes | No | - |
| 14 | 1 | 11-100 | 3 | 5 | Left | No | - |  |
| 15 | 1 | 7-100 | 2 | 3 | Left | No | - |  |
| 16 | 1 | 8-100 | 2 | 12 | Right | No | - |  |
| 17 | 2 | 8-250, 7-100 | 3 | 3 | Right | No | - |  |
| 18 | 3 | 9-100, 8-100, 8-150 | 3 | 15 | Left | No | - |  |
